# Artificial Intelligence for the Evaluation of Postures Using Radar Technology: A Case Study

**DOI:** 10.3390/s24196208

**Published:** 2024-09-25

**Authors:** Davide De Vittorio, Antonio Barili, Giovanni Danese, Elisa Marenzi

**Affiliations:** Department of Electrical, Computer and Biomedical Engineering, University of Pavia, 27100 Pavia, Italy; davide.devittorio01@universitadipavia.it (D.D.V.); antonio.barili@unipv.it (A.B.); giovanni.danese@unipv.it (G.D.)

**Keywords:** artificial intelligence, LSTM, posture analysis, radar technology, embedded systems, fall detection

## Abstract

In the last few decades, major progress has been made in the medical field; in particular, new treatments and advanced health technologies allow for considerable improvements in life expectancy and, more broadly, in quality of life. As a consequence, the number of elderly people is expected to increase in the following years. This trend, along with the need to improve the independence of frail people, has led to the development of unobtrusive solutions to monitor daily activities and provide feedback in case of risky situations and falls. Monitoring devices based on radar sensors represent a possible approach to tackle postural analysis while preserving the person’s privacy and are especially useful in domestic environments. This work presents an innovative solution that combines millimeter-wave radar technology with artificial intelligence (AI) to detect different types of postures: a series of algorithms and neural network methodologies are evaluated using experimental acquisitions with healthy subjects. All methods produce very good results according to the main parameters evaluating performance; the long short-term memory (LSTM) and GRU show the most consistent results while, at the same time, maintaining reduced computational complexity, thus providing a very good candidate to be implemented in a dedicated embedded system designed to monitor postures.

## 1. Introduction

The 2024 World Population Prospects report of the United Nations highlights that, in the coming years, the overall population will steadily increase; in fact, it is estimated that, by the year 2100, the global population will reach 10.4 billion [1]. A direct consequence of this trend is the corresponding growth in the elderly population and, in particular, in the number of frail people. Their higher degree of vulnerability, related to their age, concomitant pathologies and/or disabilities, presents the need to monitor and improve not only their health but also their everyday lives; in fact, a higher level of independence and autonomy directly contributes to better quality of life.

In order to achieve this, recent approaches have paved the way for the development of the so-called ambient assisted living (AAL) [2,3]. This field of research aims at evaluating the behavior of people, both indoors and outdoors, through the use of sensors and systems of a different nature, including non-contact devices like cameras and volumetric sensors, as well as wearables.

In the context of assuring people’s independence, the evaluation of postures has become a major objective, since frail people are at a higher risk of sudden illness and falling represents a potential issue for their health, with a non-negligible portion of people with serious injuries [4]. The promptness in detecting an anomalous situation and providing adequate assistance could make a significant difference in a person’s life; hence, it is of extreme importance to ensure the availability of systems that are capable of continuous measurement, especially when a frail person lives alone. In fact, such systems could also immediately activate caregivers and assistance in case of problems.

However, an important feature of whichever system is to be implemented, especially in a domestic environment, is the subject’s privacy. This is a source of concern, since many individuals perceive camera systems as intrusive, even though commercial tools employed for such purposes do not record the face and body of the person but only the body segments, in order to reconstruct the posture and position inside a room. An example of one of the most used systems is the Microsoft Kinect camera, designed to detect movements and posture [5]. Such devices provide good accuracy but their detection ability could be reduced in rooms with low illumination. In addition, to observe the environment with an appropriate level of detail, multiple cameras are necessary, thus considerably increasing the costs.

Another potential limitation when determining the most appropriate device concerns the use of wearable systems. In this case, the choice depends on factors not simply related to the technological appropriateness, such as the sensing principle, the battery duration (in fact, while under recharge, a wearable tool cannot be used) and the dimensions of the overall system [6,7]. In fact, the ease of use and the unobtrusiveness are also strongly influenced by the purpose and the type of user; in particular, the latter requirement is strongly dependent on each individual person. In fact, it has been observed that the compliance of users in wearing certain wearables (such as larger wristbands) was reduced due to the person being uncomfortable and her/his inner perception that other people could see the device and misinterpret its function. Another source of issues is the user forgetting to recharge the instrument.

Therefore, research has focused on studying and developing solutions that could be perceived as safe and secure from the users’ side and, at the same time, technologically robust in terms of durability and the accuracy of detection and monitoring.

Radar technology represents a solid approach in many different fields, especially in the biological one, particularly for disease detection and diagnosis, since it allows the observation of innovative properties in an unobtrusive way [8]. In addition, it is non-invasive and preserves the privacy of the subject. Therefore, in recent years, studies have been conducted in the fields of personalized patient care and monitoring and ambient assisted living [9,10,11]. In fact, a series of studies were recently published on the detection of various movements, like falls, sleep, respiration and vital signs [12,13,14]. In this regard, radar sensing technology represents an emerging field for human activity recognition and wellbeing monitoring, and it greatly benefits from machine learning (ML) and deep learning (DL) approaches to discriminate between different physiological and physical states and conditions.

Most of the solutions proposed to date are focused on a combination of different sensing modalities—for example, radar and MEMS low-resolution IR sensors or polysomnography—or a combination of devices of the same type, usually for very specific tasks (i.e., falling from a bed) [12,13,15]. The presented work aims to move a step further by evaluating a broader range of postures through the use of a single MIMO FMCW sensor.

In this context, frequency-modulated continuous wave (FMCW) radars and also multi-input and multi-output (MIMO) FMCW are becoming increasingly employed [16]. Such sensors are able to detect the body of a person moving in a room, distinguishing specific body parts (i.e., arms or legs), depending on the purpose of the measurement. This is particularly important when detecting different postures and even falls, since different devices from the FMCW family can be used, such as MIMO FMCW for gait detection [16], the evaluation of falls and activity recognition with high-frequency FMCW radar for multi-perspective micro-Doppler (μ-D) [17] and even 2D localization combined with vital signs monitoring through single-input and single-output (SISO) FMCW [18].

For the efficient study of posture and falls—and especially to reduce as much as possible the risk of wrongly detecting potentially dangerous situations—classification algorithms, as well as machine and deep learning methods, can be employed. Different methodologies can be envisioned. As an example, the recent attention mechanism, designed for natural language processing and then extended to the field of image processing [19,20,21,22,23], has now been evaluated even for human activity recognition tasks, thanks to its ability to highlight spatiotemporal relationships [24,25,26]. More consolidated approaches rely on widespread classifiers like K-nearest neighbors (KNN) and random forest, since they work well for feature extraction in both the time and frequency domains. Even support vector machines (SVM) can be applied, especially in contexts where two different classes need to be discriminated [25]. Other approaches make use of artificial neural networks or convolutional neural networks, particularly in the case of multi-sensor systems [27]. In the last few years, a methodology that has gained an important role is long short-term memory (LSTM), together with some of its implementations. In fact, in the AAL field, bi-directional LSTM has already been used to process data coming from multiple devices or directly from open-source datasets, sometimes in combination with a gated recurrent unit (GRU) network [28,29]. In particular, in the case of radar measurements, the signal to be analyzed is a time series; therefore, LSTM and GRUs represent the most appropriate approaches for this type of input, as firstly stated in [30]. In this context, our approach stems from this consideration; therefore, the ML and DL methods considered for this purpose represent an evolution compared to other previous works [14,15,31].

The aim of this work is to use MIMO radar technology to observe people in indoor environments in a continuous, unobtrusive and precise way. Since this work concerns a prototypical application, the presented tests have been designed and conducted in standard conditions, involving healthy subjects. Such a system will be provided with AI approaches—in particular, clustering algorithms and neural networks—to detect and discriminate between different postures, in order to highlight falls. In this way, it will be possible to combine the advantages of the continuous and low-cost monitoring typical of wearable devices with the precision of movement detection seen in cameras, while, at the same time, avoiding the need for periodic charges and maintaining people’s privacy.

This paper is structured as follows. The Materials and Methods section presents the radar technology used and the various AI methods tested. The Results section demonstrates the behavior of the AI methodologies in different experiments with healthy subjects, while the Discussion evaluates the most appropriate approach for the purpose of the work and introduces possible improvements that could be implemented in future developments.

## 2. Materials and Methods

### 2.1. Radar MIMO Technology

In this work, a radar sensor based on multi-input and multi-output technology has been used to observe people in a room and their corresponding movements. Radio detection and ranging (radar) technology is used to perform detection in various application fields and it is based on electromagnetic waves that allow one to identify the position and the characteristics of target objects in their fields of view. Transmission is performed through an antenna in a frequency range of 230 MHz and 110 GHz, and an object is detected based on the amount of reflected waves depending on various factors, among which are the object’s surface and dimensions. This signal is acquired by a receiving antenna and then processed to extract useful information.

Among the various types of sensors, which differ according to the specific measurement purpose, this work focuses on MIMO technology, where a single device works in the range of millimeter waves and it incorporates multiple transmitters and multiple receivers. In this way, it is possible to execute tests with a much smaller number of devices (ideally a single one in environments with limited volumes) compared to single-input multiple-output (SIMO) radars, since MIMO transducers can obtain the same resolution angle as with multiple SIMO but with a single processing chain. In fact, when employing SIMO technology, the higher the number of receiving antennas, the higher their resolution angle. However, this situation is associated with an important problem: each antenna has its own processing chain; consequently, more antennas will considerably increase the costs, processing time and power consumption, together with the size of the device. To overcome this drawback, MIMO devices are able to improve the resolution by incorporating multiple receiving (Nrx) and transmitting (Ntx) antennas; in fact, they can obtain the same resolution angle as a SIMO device, but with only Nrx processing chains, instead of the Nrx*Ntx chains required when using SIMO technology.

In this case, multiple antennae, both receiving and transmitting ones, are present, and the millimeter range is convenient for our application because smaller sensors can be used, which are also of low power and low consumption. In addition, such devices operate in frequencies in the range of 80 GHz, thus allowing for high precision in detecting movements, i.e., in the order of fractions of millimeters.

The device adopted is the IWR6843AOPEVM model [32], produced and commercialized by Texas Instruments. Its functioning principle is based on the Compressed High-Intensity Radar Pulse (CHIRP) signal, which is part of the family of frequency-modulated continuous-wave radar, with frequencies linearly increasing with time. The board is an antenna-on-package (AoP) module that integrates also a wide-field-of-view (FoV) antenna, and it has the advantage of providing direct access to the point cloud data. It also comprises a USB interface and it is equipped with a millimeter-wave sensor working in the range [60–64 GHz], together with four receiving and three transmitting antennae, with 120° for both the azimuth and elevation fields of view. Such a device allows us to combine the advantages of MIMO technology working in the millimeter-wave range, thus resulting in a compact sensing tool, able to simultaneously detect numerous different movements while maintaining low consumption.

**Figure 1:**
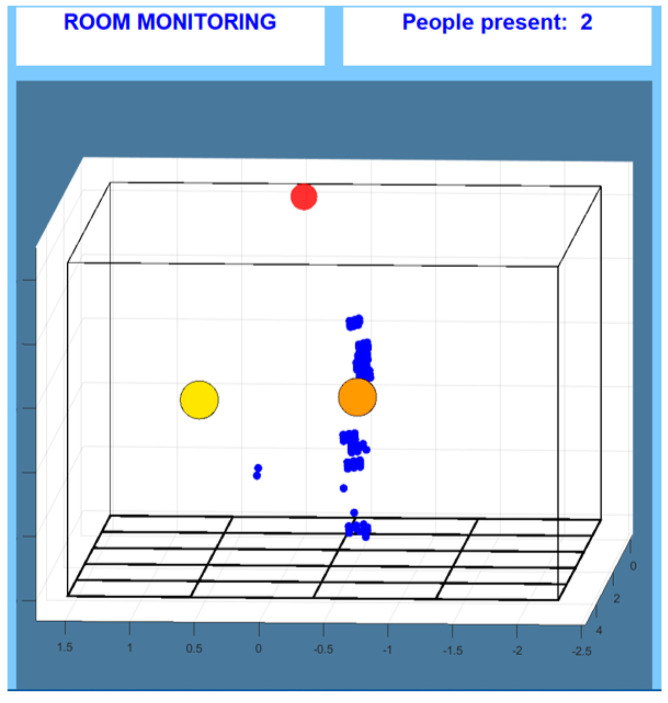
Visual output: the red circle represents the sensing device and shows its positioning in the volume under monitoring; the yellow and orange circles are the spots indicating that two people are in the room, while the blue small circles form the point clouds. In this image, only the person on the right (orange spot) has a large and well-defined point cloud, while the person identified with the yellow spot has only a few points in the cloud.

For the proposed solution, a single board has been employed and tested in different rooms, with different configurations. Here, the output of the board undergoes initial pre-processing to clean the signal through proprietary software. This output signal, as shown in Figure 1, represents the input to our posture detection strategy, and it is composed of the point cloud (consisting of a series of small blue circles) and a so-called spot, which is graphically represented by a sphere with a larger diameter and a different color compared to the points in the cloud. This element identifies the person, and it is designed to be shown approximately at the level of the upper torso (usually where the person has a larger area of reflection to the sensor). A single spot is formed for every person in the volume under analysis; during an acquisition, and also successively offline, either the spot, the point cloud or both can be visualized. Moreover, the three spatial coordinates of every point in the cloud and the spot are stored, for every instance, and are available for further processing and analysis. The device allows us to set the dimensions of the volume to be considered as a configuration parameter, in order to customize it to different rooms and even to avoid objects that could produce noise during measurements.

### 2.2. Artificial Intelligence Approaches

Various ML and DL methodologies have been evaluated, taking into consideration the accuracy in detecting diverse postures and falls but also their computational loads. In fact, in this context, it is not possible to consider only the performance of the algorithm; its computational load also needs to be taken into account, since this is a device conceived for Internet of Things (IoT) applications. Hence, the final monitoring system will have the characteristics of an embedded system; therefore, the resource usage (in terms of the battery and execution time, which are the most critical aspects) needs to be thoroughly evaluated, as this could greatly influence the choice of the most suitable classification method.

Consequently, two approaches have been followed: the first one consists of the application of three of the most important and common classifiers, thanks to their low computational cost; the second adopts a series of deep learning techniques, specifically in the broad family of recurrent neural networks (RNN), namely the long short-term memory methodology, which has been tested using different configurations and implementations, and a GRU method has also been employed. Their choice is motivated by the potential benefits in terms of detection accuracy, and, even though a higher computational cost is expected compared to the classification algorithms, its newest implementations require fewer resources and they therefore can be considered good candidates.

The clustering algorithms employed are KNN, random forest and SVM. The DL approaches used, instead, are the GRU methodology and the LSTM in the following three implementations: traditional LSTM, Bi-LSTM and Projected LSTM.

#### 2.2.1. K-Nearest Neighbors

The KNN approach is one of the most straightforward classification algorithms, even in its basic implementation [33]. In fact, it allows the classification of a specific element based on its K-nearest points in the training dataset. Such points are determined based on the spatial distance and the principle is the following: if the majority of the training points close to the one examined belong to a certain category, then even the same examined point will belong to the same category. In AAL, it has been applied in various applications, including fall detection, sometimes in combination with SVM, but also to discriminate between various daily activities [34,35]. The mathematics at the foundation of this algorithm consists of the following equations, used to calculate the Euclidean distance between two points in space. When all such distances have been calculated, the minimum is determined and the corresponding class is selected:

(1) $$C=\min\left(d_{i}\right)\;d_{i}=\sqrt{{\left(x_{2}-x_{1}\right)}^{2}+{\left(y_{2}-y_{1}\right)}^{2}+{\left(z_{2}-z_{1}\right)}^{2}}$$

where *C* represents the output class, *d_i_*, with *i* in the range [0;N] and N as the number of training elements, is the Euclidean distance in three dimensions, and *x*, *y* and *z* are the spatial coordinates of the points.

#### 2.2.2. Random Forest

This learning algorithm is based on decision trees, each developed from a random subgroup of training data. When an element needs to be classified, it will be processed by all trees and give its own output category through the *entropy* indicator. The final classification will be the modal value among all trees [34].

(2) $$entropy=\overset{C}{\underset{i=1}{\sum }}\left(-p_{i}\right){log}_{2}p_{i}$$

where *p_i_* is the frequency of a single class, while *C* is the number of classes.

#### 2.2.3. Support Vector Machines

The basic implementation of SVM is designed to operate on only two classes, since its aim is to determine the hypothetical plane separating them in space with the maximum accuracy [36]. In our implementation, we focused only on the distinction between a fall and the standing posture.

(3) $$wx_{i}+b\geq 1\;for\;y_{i}=1\;wx_{i}+b\leq -1\;for\;y_{i}=-1$$

Combining these equations, the result is the following:

(4) $$y_{i}\left(wx_{i}+b\right)-1\;\geq 0\;for\;y_{i}=1;-1$$

#### 2.2.4. Long Short-Term Memory and Gated Recurrent Unit

The LSTM methodology is part of the broader family of RNNs and it is usually applied to learn long-term temporal dependencies [37]. The only difference is that the input is distributed over four gates, namely the input, output, cell and forget.

**Figure 2:**
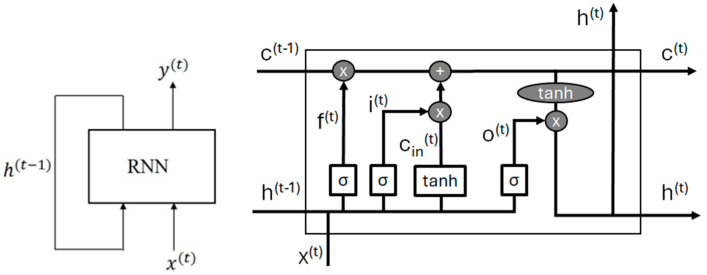
RNN principle of functioning and architecture of the LSTM cell.

In this context, a stacked LSTM (Figure 2) has been firstly employed [38]. It comprises a fully connected layer to process the inputs, whose output is given as input to a rectified linear unit (ReLU) function, namely the LSTM cell, and one last fully connected layer for the outputs. Its output represents the input to the *softmax* function, thus providing the final output.

The mathematics behind this approach is the following:

(5) $$i^{\left(t\right)}=sigmoid\left(W_{i}x^{\left(t\right)}+U_{i}h^{\left(t-1\right)}+b_{i}\right)\;o^{\left(t\right)}=sigmoid\left(W_{o}x^{\left(t\right)}+U_{o}h^{\left(t-1\right)}+b_{o}+b_{forget}\right)\;c_{in}^{\left(t\right)}=tanh\left(W_{c}x^{\left(t\right)}+U_{c}h^{\left(t-1\right)}+b_{c}\right)\;f^{\left(t\right)}=sigmoid\left(W_{f}x^{\left(t\right)}+U_{f}h^{\left(t-1\right)}+b_{f}\right)\;c^{\left(t\right)}=f^{\left(t\right)}*c^{\left(t-1\right)}+i^{\left(t\right)}*c_{in}^{\left(t\right)}\;h^{\left(t\right)}=o^{\left(t\right)}*tanh\left(c^{\left(t\right)}\right)$$

where $W,U\in {\mathbb{R}}^{LS\times LS}$, $i^{\left(t\right)},\;\;o^{\left(t\right)},\;c_{in}^{\left(t\right)},\;f^{\left(t\right)},\;c^{\left(t\right)},\;h^{\left(t\right)},\;x^{\left(t\right)},\;b\in {\mathbb{R}}^{LS}$, and the operator ∗ stands for the element-wise product. $b_{forget}$ is the forget bias, typically equal to 1, while LS is the *inner size* (or *LSTM size*) hyperparameter.

The network has seven layers.

1. *Feature Input Layer*: The network input that checks whether data is in the correct format.
2. *Fully Connected Layer*: Each neuron is connected to all neurons of the previous layer. The objective is to detect non-linear relationships between the input data characteristics, to better understand the input data relations.
3. *ReLU Layer*: The output of the first fully connected layer is the input to the *f*(*x*) function, the ReLU. This layer introduces non-linearity in the model, allowing the network to train and model complex relations among the data:
  (6) $$f\left(x\right)=\max\left(0,x\right)$$
4. *LSTM Layer*: This manages information related to time series, since it is designed to detect and keep long-term dependencies between temporal data, thus improving the network’s ability to make predictions based on previous instants.
5. *Fully Connected Layer*: The second layer of this type, it converts the output of the previous layer into a vector with dimensions equal to the number of output classes, and it is used to make the final predictions, preparing the data for classification.
6. *Softmax Layer:* This function converts the output of the previous layer into a probability vector by calculating the probability that each value belongs to one of the possible postural categories.
7. *Classification Layer*: The last layer, it determines the most suitable option considering the obtained probabilities. The network uses such information to improve its prediction capabilities.

**Figure 3:**
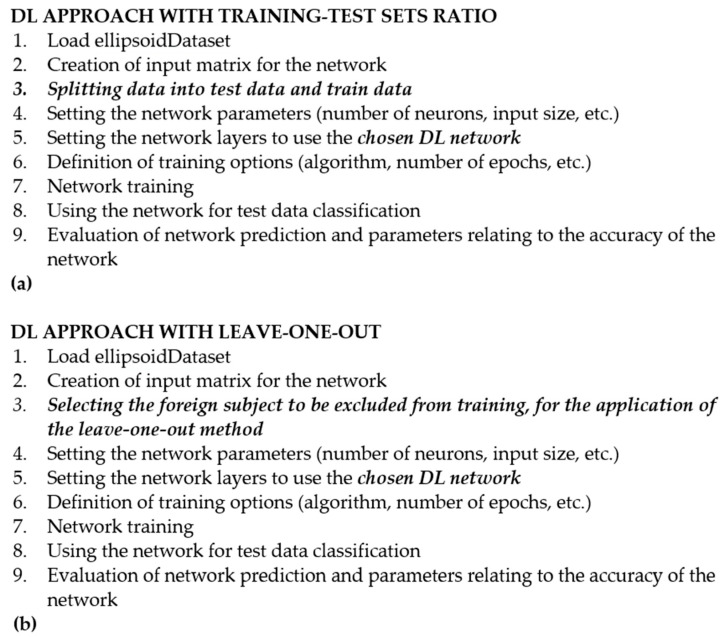
Pseudo-code for LSTM, Bi-LSTM, projected LSTM and GRU, in the case of the subdivision between the training and test sets (**a**) and for the leave-one-out approach (**b**). Line 6 differs in the two cases, since, in (**a**), there is the subdivision into training and test sets with all ratios previously mentioned; in (**b**), instead, it considers the single subject left out from the training, following the leave-one-out approach. Line 8, in addition, has been written in a generalized way since it depends on the DL approach considered (written in italics, i.e., LSTM).

Together with this method, a Bi-LSTM, a Projected LSTM and a GRU algorithm have been applied using MATLAB (version R2023b), since all of them have shown promising results for fall detection and therefore they could be evaluated even for the broader field of posture detection [39,40]. Figure 3 shows the pseudo-code for the above-mentioned approaches.

The reason for implementing this type of approach is linked to the RNN’s capability to retain a memory of the previous steps and its satisfactory behavior when dealing with temporal series.

## 3. Results

**Figure 4:**
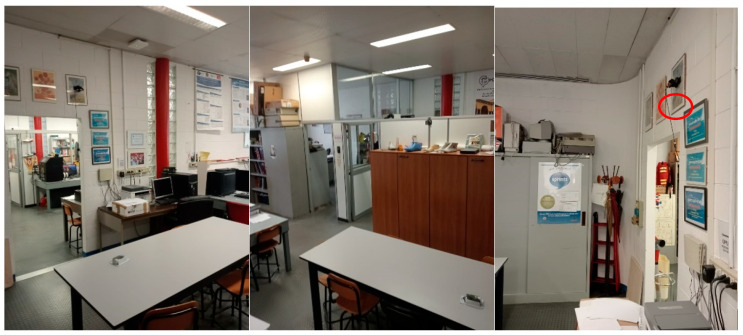
Room where the first experimental tests were performed, shown from different angles. The device can be seen in the top-right corner of the third image, on the right of the page (highlighted by the red circle).

The system has been tested in different indoor environments. At first, it was placed in a laboratory room with dimensions of 4 × 3 m^2^ at a height of 2.5 m and inclined at a 45° angle (Figure 4). Both the angle and height were kept the same during all experimental tests. The aim of the first evaluation was to define appropriate values for the configuration parameters that could be kept stable independently of the room.

**Figure 5:**
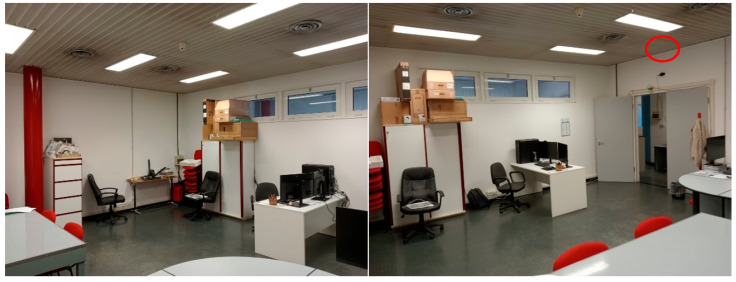
Second room, where all other experimental tests were performed, shown from different angles. The device can be seen in the top-right corner of the second image, on the right of the page, stuck to the wall over the door (highlighted by the red circle). Since, here, there was more room for movement, walking, sitting and falling tests were conducted.

After this, tests were conducted in another room to give more freedom of movement to the subjects (Figure 5); the dimensions were 4 × 5 m^2^, with the height and angle of the sensor kept the same as in the previous room.

**Figure 6:**
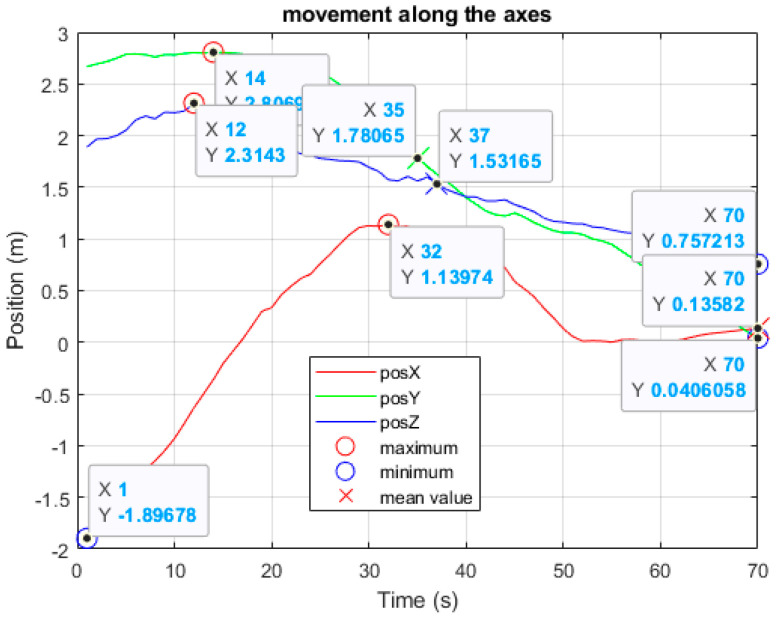
The figure shows a person randomly walking in the room. The graph shows the 3 spatial coordinates (x in red, y in green and z in blue), with their maximum (red circle), minimum (blue circle) and mean values (red cross). As can be seen, the z coordinate is reduced when the subject moves closer to the sensor, as shown by the other two coordinates, x and y, having smaller values as well.

It can be observed that the height at which the spot is formed is directly proportional to the distance from the sensor; therefore, a person that is located farther away will have a spot at a higher z coordinate (Figure 6). This is due to the fact that, in this case, the entire body of the subject will be detectable, while, in places closer to the device, it is more difficult to identify the correct height.

**Figure 7:**
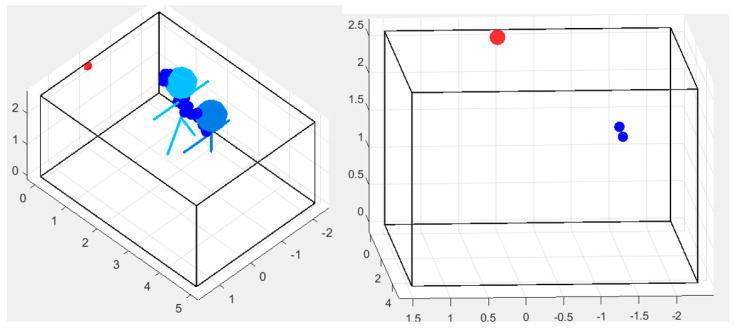
The image on the left displays a distortion in the point cloud and also a double spot, which could be mistaken as indicating two people in the room. The schematic body reconstruction clearly highlights that, without prior knowledge of the measurement context, the situation could be easily wrongly interpreted. The three-dimensional representation on the right shows that, even with no one in the room, metallic furniture produces reflections, resulting in an actual (albeit small) point cloud.

Moreover, when metal objects or furniture are present in the evaluation volume, they cause distortions in the measurements, as shown in Figure 7. In particular, this is evident when the person comes near such elements, since the point cloud suddenly alters its shape in an unrealistic way and an additional spot appears, representing an artifact. The solution for our case was to reduce the volume by eliminating any critical objects. Since this approach is not feasible in all situations, a solution in such conditions would be to add a second sensor placed in a different part of the room, to provide redundancy to the measurement and detect unrealistic spots and point clouds.

To evaluate various postures and movements, different approaches have been studied in order to determine the most appropriate one for this scenario. All tests described in this paper involved only a single subject in the room, to reduce possible sources of noise and misinterpretation by the system when analyzing the results.

**Figure 8:**
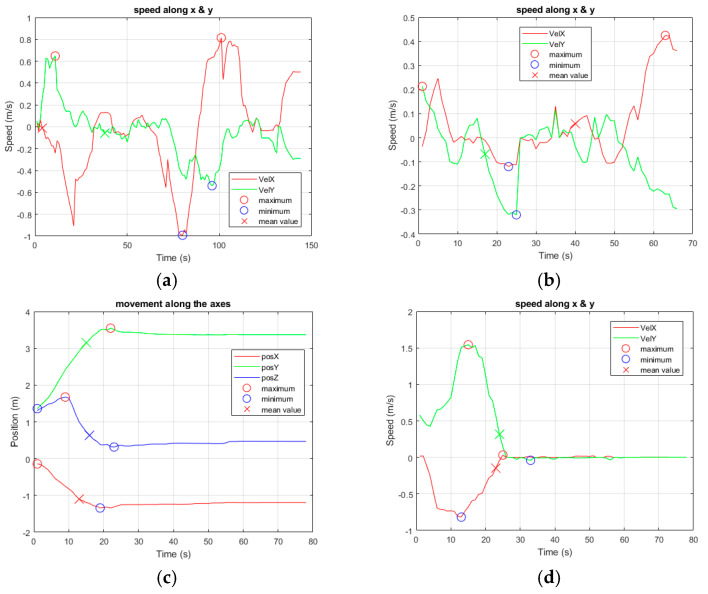
The figures show the speed on the two axes, x and y, related to a person randomly walking in the room in (**a**,**b**), and falling in (**c**,**d**). The crosses always represent the mean value of the corresponding curve. In (**c**), the position along the three axes is reported, and, in (**d**), the speed of the fall is observed. In this case, the legend of colors and indicators is the same as in Figure 6. Compared to walking, a fall presents a very rapid increase in speed, followed by a prolonged stop.

First of all, the movement speed was evaluated during walking vs. falling tasks: a healthy subject was asked to walk randomly at a normal speed in the room and successively to fall. It was noted that the speed range while walking was always within ±1 m/s, whereas an acceleration was observed during the fall (Figure 8).

**Figure 9:**
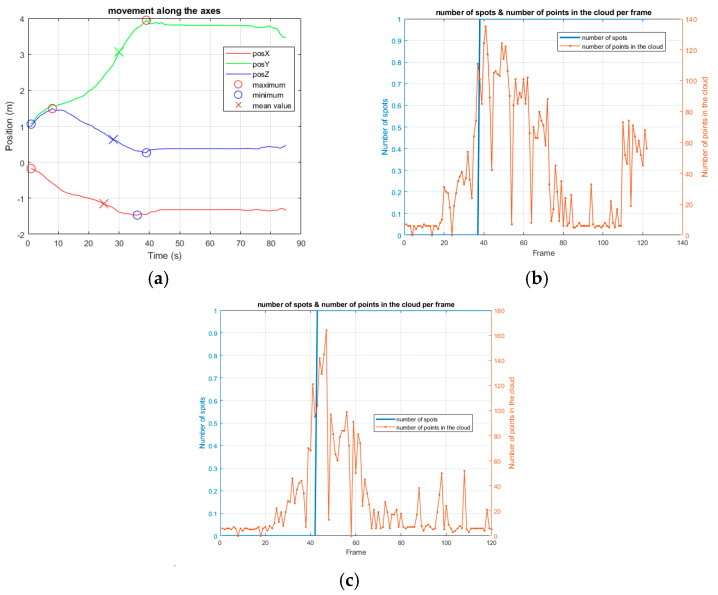
(**a**,**b**) show the same test as presented in Figure 8c,d, while (**c**) presents another experiment of a person falling. The crosses always represent the mean value of the corresponding curve. The movement graphs are associated with the corresponding spot and number of points in the cloud for each frame. The device works by collecting 10 frames/s. In both cases, the evolution is very similar, as can be derived in (**b**,**c**), respectively. Here, the person is identified by the system after a short transient period according to the blue line. The number of points in the cloud is given at any frame by the orange graph and clearly shows that, after the person is detected, the number decreases, and it is considerably reduced when the fall occurs, potentially causing problems in reconstructing the point cloud.

However, in this latter case, an issue occurs at the end of the movement: since it is very important to determine if the person is well or injured, a traditional approach is to measure the movements seen after falling, but, if the person has been able to dampen the fall (i.e., by grasping furniture), a speed variation cannot be adequately observed. This could potentially introduce bias in the detection procedure and lead to the misinterpretation of a dangerous situation. Therefore, this parameter cannot be considered as the sole indicator to discriminate movements and risky situations, and more accurate methodologies need to be developed. In this regard, relevant observation needs to be performed concerning the number of points in the cloud, since a reduction occurs at the moment of the fall. This could be explained by the person suddenly, but involuntarily, distancing her/himself from the sensing element, producing a significant variation in the point cloud (Figure 9).

In fact, since the radar sensor emits a conic bundle of chirp waves, when the subject is closer to the device, more reflections occur, but the precision in determining the real dimensions of the body is lower (since the aperture angle is still small). In contrast, when the person is farther from the sensor, fewer waves are reflected from a larger area, hence being more representative of the real body shape.

In this context, a series of tests on healthy subjects was performed while they were switching positions between upright, walking, sitting and falling. The working frequency of the instrument is 10 frames/s. This allows for the accurate detection of even small posture changes.

When evaluating the fall, the experiment consisted of a person doing a short walk and then falling. Twelve tests were performed by different subjects and the following parameters were considered:

- The speed, taking into account also the maximum peak reached during the fall, which can be positive or negative depending on its direction;
- The mean speed immediately after the fall, since, usually, the person does not move, or they move very little in the first few instants after falling, especially among the elderly;
- The z coordinate, which is the most significant indicator, since it is considerably reduced when a fall occurs.

In these first tests, only the spot has been evaluated and the first derivative of the z coordinate has been calculated to detect its point of maximum negative variation, since it represents the instant when a fall occurs. Once this point has been determined, 40 frames around it (corresponding to the 2 s before and after the minimum z value) are considered to analyze the speed during such a time interval, because this is the time observed for a person to fall from standing upright.

The post-fall speed has been calculated considering the interval starting from the 10th to the 50th frame, after obtaining the maximum negative variation in the z coordinate.

Such values are saved in a file and given as input to the algorithms described in the previous section according to a series of different strategies performed for all AI methods developed:

a. 50-50 ratio, where half of the data, mixing all data coming from all subjects, are used for training and the remaining half for the test;
b. 60-40 ratio, where 60% of the data, mixing all data coming from all subjects, are used for training and the remaining 40% for the test;
c. 70-30 ratio—in this and the following two cases, the datasets’ division is the same as in the previous point;
d. 80-20 ratio;
e. 90-10 ratio;
f. leave-one-out, where a single experiment with one subject is used as a test and all other tests represent the training set.

To detect the other postures, different people carried out two additional sets of experiments, for a total of 14 tests.

1. Entering the room and sitting on a chair positioned at 2.5 m from the radar sensor, followed by standing up and returning to the door—this sequence of movements was performed five times.
2. Walking toward the same chair as in the previous test while maintaining a fixed *y* distance from the sensor, followed by sitting down, standing up and returning to the starting point. Even in this case, the sequence was repeated five times. Such a test was performed also to evaluate the posture while keeping both the number of points in the cloud and the height of the spot stable.

All methodologies have been used both considering the three experiments separately and the combination of the last two datasets, thus doubling the data available for training.

**Figure 10:**
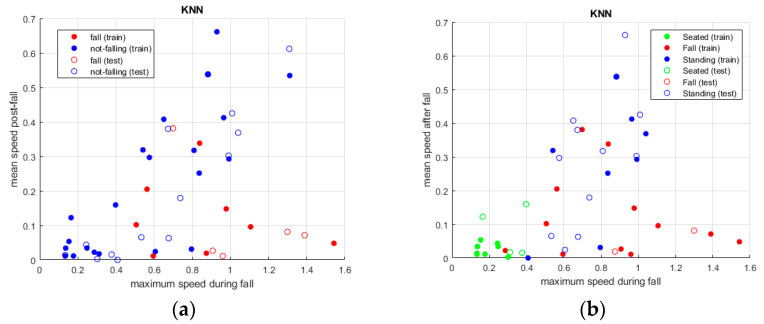
(**a**) is the graphical representation of two classes of output, standing and falling, where the colored circles are those from the training set, while the others are the detected ones. (**b**) is the same representation with the addition of the sitting posture.

**Figure 11:**
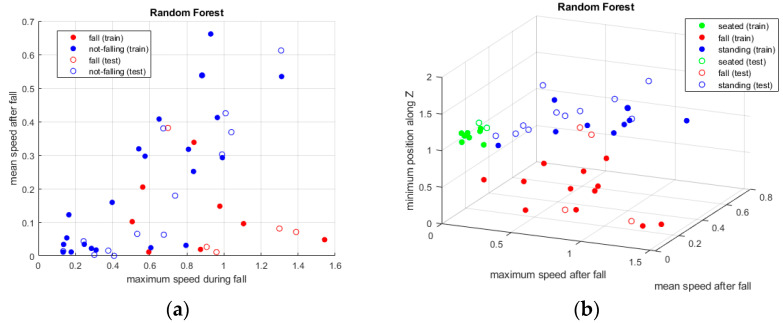
Same representations as in Figure 10. Here, the classes appear more separated compared to the previous method, but the results are very similar.

**Figure 12:**
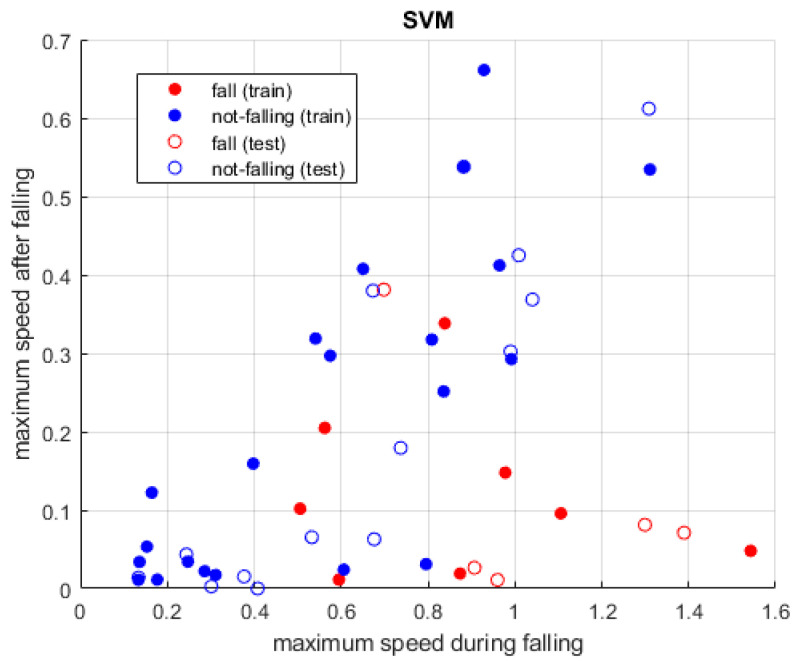
Two-class detection was performed between falling and standing upright. As in the previous approaches, the behavior of the algorithm is good and it allows one to discriminate between postures.

The three classification algorithms (KNN, random forest and SVM) have been applied to the three datasets, as described in the previous subsection, and Figure 10, Figure 11 and Figure 12 show the results. When using the first dataset of people falling, two classes are evaluated, while, for the other tests, all three classes—and consequently all three datasets—are considered.

### Classifiers

KNN and random forest classifiers allow to simultaneous evaluation of all three postures, whereas in the case of SVM only binary classification has been performed between the standing and falling postures in the first set of tests. In fact, although a multiclass SVM could be applied, the accuracy of this algorithm is comparable to that of the previous ones (as shown in Table 1), with the drawback of being always more computationally demanding than KNN and random forest classifiers. This is true if the multiclass option is chosen but also if applying multiple SVM in series to evaluate all pairs of postures. As a consequence, in our specific context, SVM has comparable results regarding the classification accuracy but lower performance in terms of the computational cost. Therefore, it does not represent the most suitable approach. KNN instead is confirmed as the best algorithm, with only slightly better results than random forest.

All three methodologies showed very good results, with values above 90%, and KNN was the best approach. It is followed by the random forest classifier, with a lower but very similar value, while SVM presented a slightly worse output.

As previously mentioned, the sensing device can provide both the spot that identifies the person in the room and the point cloud, giving an idea of the volume of the body. In the analysis with KNN, random forest and SVM, a single spot was considered to determine and discriminate the various postures.

**Figure 13:**
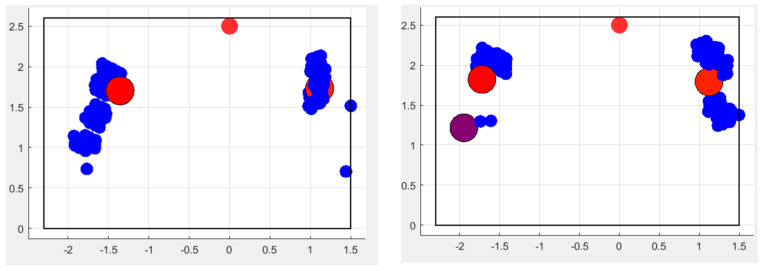
The images show two people in the same room that are in an upright position and periodically walk. On the left, the software correctly detects both of them, each with a single spot and corresponding point cloud. On the right, the image presents one subject with two associated spots, of which only the red one is correct, while the purple circle is an artifact.

Even though good performance was obtained, a non-negligible issue that arose was the incorrect identification of the subject using the spot. More specifically, depending on the distance between the subject and the radar sensor, sometimes, two spots were produced by the software. This could pose a major problem regarding the misclassification of the number of people in the room, as well as their posture. In fact, the additional spot originated from an entirely different part of the body, such as at the knee level (instead of the upper torso), leading to the misinterpretation of a real upright stance as a fall (Figure 13). This can happen when the sensor has too few points in the cloud and therefore reconstructs an overly small or dispersed volume; hence, the software automatically separates the volumes and produces two spots, each associated with one of the smaller point clouds. This is due to the functioning principle of the radar sensor, which reconstructs the volume based on the quantity of reflections associated with a subject. The volume dimensions are chosen based on the real volume of a person’s body and her/his typical movements and abilities, and, in this case, the width and the depth of the volume were too small. Therefore, this issue was addressed by analyzing the configuration parameters of the sensor (available for free on the TI website [32]).

**Figure 14:**
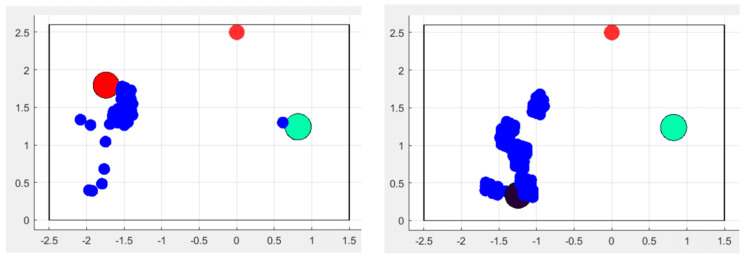
The image shows two people in a room, where the one on the left suffers from an artifact: the system loses the detection of the person for a few frames, and, when it recovers (image on the right), the reconstruction is altered towards the floor with the spot created at a very low level, which is incompatible with a person standing.

Additionally, sometimes, the system does not detect a person during all frames and fails to achieve spot detection for a few frames, while successively reconstructing it again at a much lower height (i.e., at the level of the lower legs, as in Figure 14). Again, this could lead to the misinterpretation that the person as fallen, while, in reality, she/he is still walking. The reason for this is linked to the sensor favoring movement components rather than static postures, to avoid mistaking a person as an object or piece of furniture. In this case, though, the legs are erroneously considered the most reflective part of the body and thus the identification of the subject is associated with this body portion, producing incorrect posture classification. As in the previous situation, adapting the configuration parameters has considerably reduced this problem.

**Figure 15:**
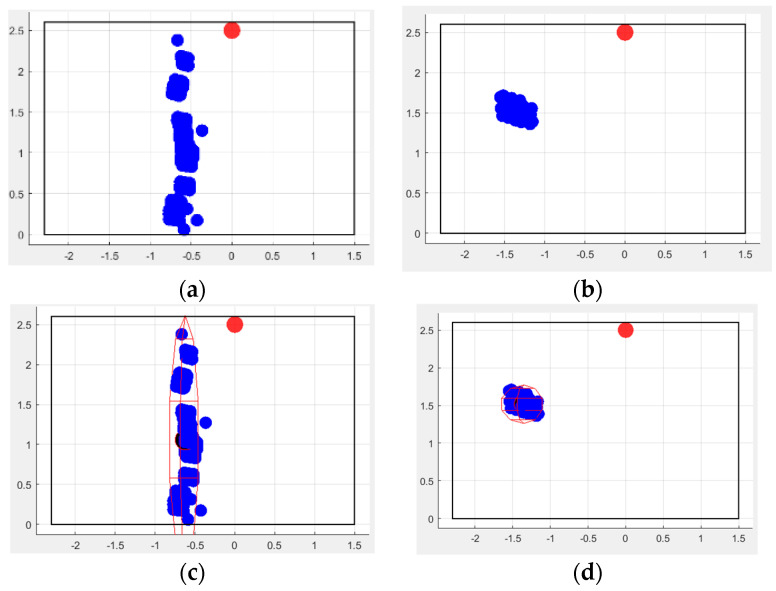
A single person standing (**a**,**c**) and sitting (**b**,**d**): in the second case the point cloud is compacted to the most reflecting part of the body, the upper torso. This is the reason that the concentration of points is localized higher than the center of gravity of the person. (**c**,**d**) show also the corresponding confidence ellipses, with very different shapes and eccentricities, since, for the seated position, it resembles a circle.

To better compensate for such artifacts, which could still occur even after the presented modifications, we decided to evaluate posture classification by combining both the position of the spot and that of the entire point cloud. This approach was followed because a significant difference between the shape of a person standing upright compared to someone seated (Figure 15a,b) can be observed.

To do this, we reproduced the morphology of the point cloud through a confidence ellipsoid and studied its shape and eccentricity when taking on different postures (Figure 15c,d). In fact, the reconstruction of a series of points with peculiar morphologies into simplified shapes (in two or three dimensions) has been performed in various application fields, for very different purposes [41,42,43]. The general aim is to have a few very straightforward parameters indicative of a property that is useful to measure or is very easy to evaluate, in order to reduce errors.

**Figure 16:**
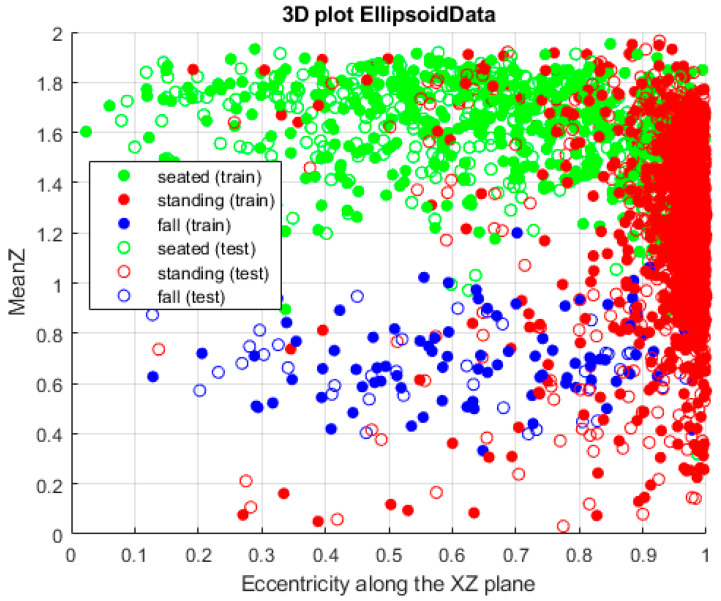
Output of the LSTM method considering the three postures. Sitting is shown in green, the fall is shown in blue and the upright position is shown in red. As above, the training sets are denoted by the fully colored circles, whereas the others denote the test sets.

In our case, the reconstruction of the point cloud stems from [44,45] and the eccentricity is computed using the following formula:

(7) $$e=\sqrt{1-\frac{b^{2}}{a^{2}}}$$

where *a* and *b* represent two semi-axes of the 3D ellipsoid along the 2D planes (XY, XZ, YZ). The more similar the lengths of the semi-axes, the more closely the value of *e* is to zero and the more the shape of the ellipsoid resembles that of a sphere. This is a fundamental indicator of different postures, since it is able to discriminate postures in a very straightforward way (as can be seen in Figure 16): a person standing will have a high eccentricity value, whereas, while seated, this parameter will change considerably. In particular, when a person shifts their posture between sitting and standing, the eccentricity along the *z* axis is the most affected one.

An interesting consideration arises concerning the *z* coordinate and the entire volume: when the person sits down, her/his volume shifts at a higher level. This seems contradictory compared to the reality of a person that is in an overall lower position; however, it is not an error because the point cloud automatically compacts itself at the location of the upper torso (as shown in Figure 15). Thus, it is compliant with the regular behavior of the employed radar sensor. The same does not happen in the case of a fall, when the z coordinate moves closer to zero.

Regarding the LSTM, Bi-LSTM, projected LSTM and GRU approaches, in order to easily compare the results, the same parameters have been used: the three coordinates of the center of the ellipsoid, its volume and the eccentricity along the three spatial planes.

The initial number of epochs was 30, but a second evaluation using only 10 epochs produced comparable results; therefore, it was decided to keep the latter number. During each of them, the data were mixed to improve the generalization and avoid training according to the order in which such data appear.

The tests involved all experiments performed considering the three postures: standing upright, sitting and falling.

Figure 16 clearly shows three different clusters, each associated with one of the postures considered in this work, with the network producing very good results in terms of the accuracy, which reaches a value of 0.9684. In fact, the evolution of the accuracy and the loss shows that the first quickly improves, and, after only two epochs, already reaches 90%; at the same time, the loss steadily decreases to a final value of 0.1. This is not the only performance parameter considered; in fact, all of the main indicators have been calculated, as shown in detail in Table 2, Table 3, Table 4, Table 5 and Table 6:

- Accuracy;
- Precision;
- Recall;
- F1 score;
- Area under the curve.

In particular, to discriminate between the upright position and the other two postures, it is sufficient to observe the *z* coordinate, since it always has higher values compared to the other cases. Even the volume of the point cloud shows variations, but on a smaller scale. On the other hand, to distinguish the seated posture from a fall, the most useful parameter is the eccentricity, as, in such a case, the shape of the person changes. In fact, as previously mentioned, while seated, the point cloud reaches an almost circular shape; instead, when a person falls, such a compact volume is typically not maintained and often it is an ellipsoid with high eccentricity, mainly in the XY plane. The *z* coordinate also shows variation when falling, but it is usually not significant enough to properly highlight such a condition.

Similar results can be observed also for the Bi-LSTM, projected LSTM and even GRU implementations. For all methodologies, all six training approaches have been applied, as shown.

**Figure 17:**
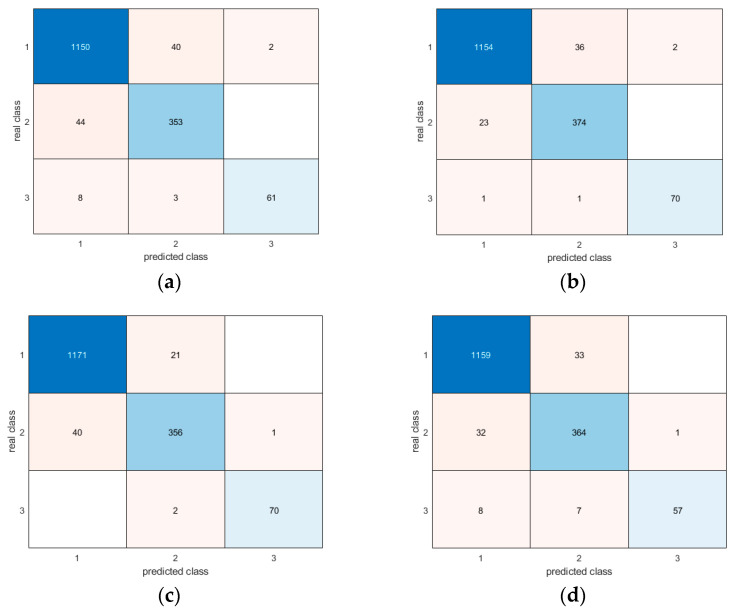
Confusion matrices for all AI methods considering all postures for the 50-50 ratio between the training and test sets: (**a**) LSTM; (**b**) Bi-LSTM; (**c**) projected LSTM; (**d**) GRU.

**Figure 18:**
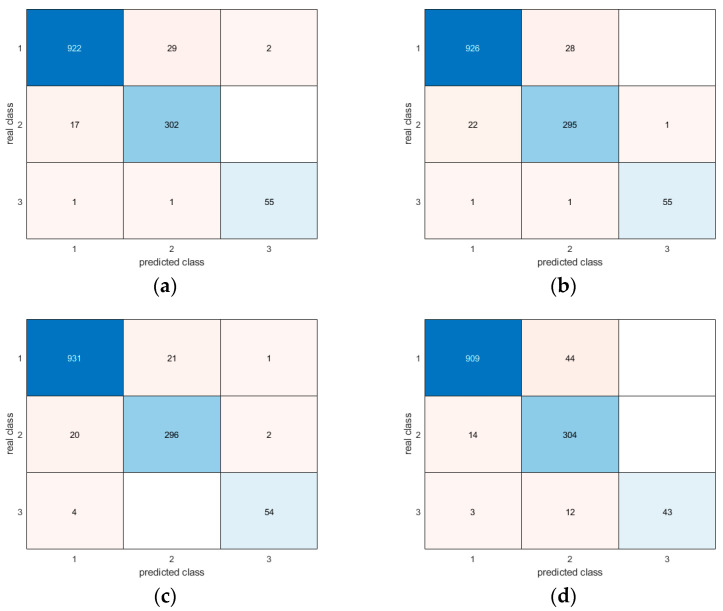
Confusion matrices for all AI methods considering all postures for the 60-40 ratio between the training and test sets: (**a**) LSTM; (**b**) Bi-LSTM; (**c**) projected LSTM; (**d**) GRU.

**Figure 19:**
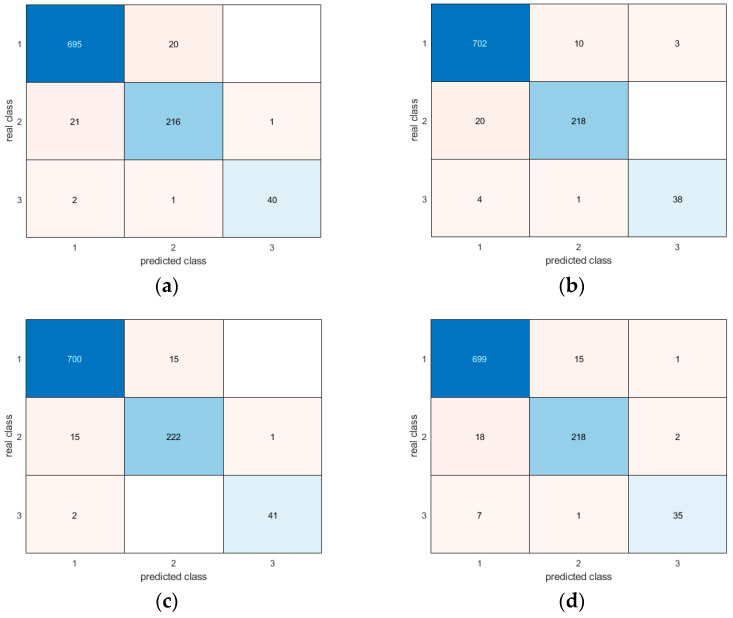
Confusion matrices for all AI methods considering all postures for the 70-30 ratio between the training and test sets: (**a**) LSTM; (**b**) Bi-LSTM; (**c**) projected LSTM; (**d**) GRU.

**Figure 20:**
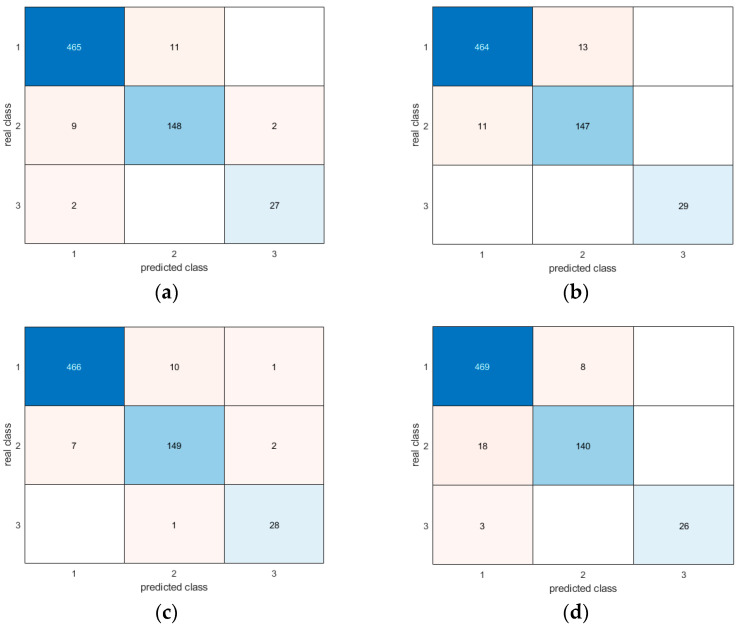
Confusion matrices for all AI methods considering all postures for the 80-20 ratio between the training and test sets: (**a**) LSTM; (**b**) Bi-LSTM; (**c**) projected LSTM; (**d**) GRU.

**Figure 21:**
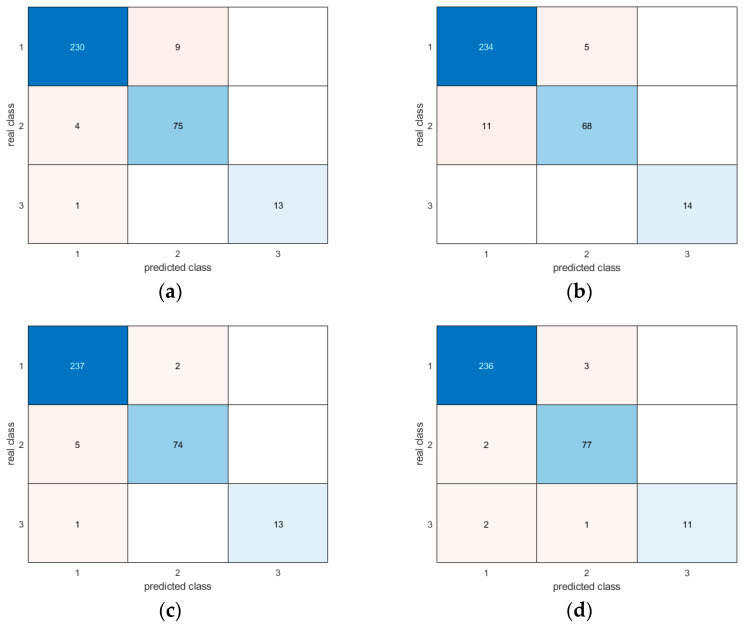
Confusion matrices for all AI methods considering all postures for the 90-10 ratio between the training and test sets: (**a**) LSTM; (**b**) Bi-LSTM; (**c**) projected LSTM; (**d**) GRU. As is clearly shown, the results are very promising, with slightly better performance in the cases of LSTM and Bi-LSTM.

**Figure 22:**
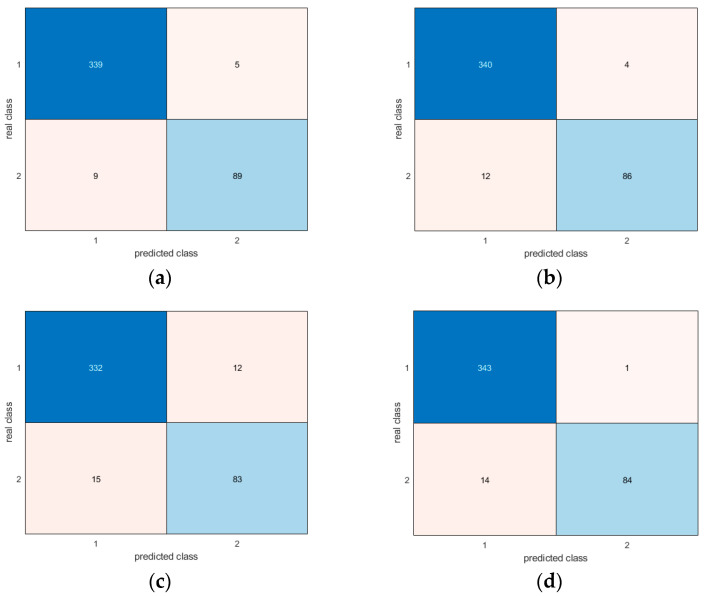
Confusion matrices for all AI methods considering only seated and upright postures: (**a**) LSTM; (**b**) Bi-LSTM; (**c**) projected LSTM; (**d**) GRU. In this case, the results are even more comparable than in Figure 17, possibly because a person lying on the floor after a fall does not assume a precise posture, while sitting and standing are more stable positions.

Moreover, the confusion matrices provide evidence of the similar and very good results for all methodologies in correctly detecting different postures (Figure 17, Figure 18, Figure 19, Figure 20, Figure 21 and Figure 22). In particular, the classes are named as follows:

1. standing upright;
2. sitting;
3. falling.

Thus, during the tests, the most probable misclassification happens between classes 1 and 2, as can be noted in Figure 17, Figure 18, Figure 19, Figure 20, Figure 21 and Figure 22, but the same situation is found also for the other ratios. However, as shown in the figure, the numbers are very small—more specifically, in the percentage range [0.3–2.7%]. In addition, even if the GRU misclassifies more classes compared to the other methodologies, the overall misclassification error is much smaller than that of the other methods, thus resulting in this being the best approach, together with the LSTM.

The computational load has also been evaluated: the neural networks follow the same sequential process. Moreover, the MATLAB tool *networkAnalyzer* provides useful insights. For every DL method, 10 epochs were run, each comprising average iterations per epoch, irrespective of the AI approach considered, according to Table 7.

The elapsed time for the entire procedure’s execution is always between 6 s (with the 50-50 ratio) and 11 s (with the 90-10 ratio), with a stable learning rate of 0.001, independent of the ratio between the training and test sets.

## 4. Discussion

The presented work examines a case study for the evaluation of different postures using radar technology.

The identification of risky situations in the elderly population, especially in domestic environments or other indoor conditions, is extremely important to avoid injuries or activate prompt assistance in the case of problems or sudden illnesses. To overcome the issue of privacy and equip the room with unobtrusive, low-cost and low-consumption devices, an innovative solution is to adopt radar sensors, such as a MIMO FMCW device, to detect the upright and seated positions, as well as falls. Due to their particular characteristics, these solutions allow the use of a very limited number of devices compared to cameras, wearables and other radar technologies, since they incorporate multiple receivers and transmitters in a single instrument (as mentioned in the Materials and Methods).

In this work, a single device based on MIMO FMCW radar technology has been evaluated and chosen for the study of different postures and the identification of falls. The signal output from the instrument has been analyzed in order to highlight both the most relevant parameters and the most appropriate classification approach for posture detection. Several classifiers and deep learning methodologies have been tested: among them, the LSTM and GRU showed the best results in discriminating between falling, standing and sitting.

This paper represents the first use case to evaluate a specific device for fall and posture detection. To this aim, only healthy subjects were enrolled in the experiments, with a consequently limited number of tests performed, in a specific type of room. Despite these limitations, the results demonstrate that a single sensor is able to provide an accurate evaluation of a person in a room and is suitable for applications in in-home monitoring. To achieve this, the output of the sensor has been analyzed by employing all of the most appropriate methodologies in the literature, considering both classification algorithms and deep learning methods. The rationale behind this approach was to determine the most suitable classification method (or methods) for this specific sensing technology in the AAL field, using a single device per room, to minimize the instrumentation employed. This could pave the way to further experiments with a larger number of healthy subjects and subsequently the inclusion of frail people, not only in a laboratory environment but also in real-life settings.

To allow for a fair comparison between the results among the three classifiers and the DL methods and guarantee the robustness of the results obtained, the training and test data have been divided, as explained in Section 3, and have been kept the same for all tests performed.

The results highlight that all methodologies give very good results, particularly the deep learning approaches. More specifically, the LSTM and GRU have the best performance, although the projected LSTM and Bi-LSTM methods have slightly different results. This is true considering both the posture detection accuracy and the computational loads of the various approaches. In fact, as shown by the output of the MATLAB functionality *networkAnalyzer*, the footprints of the various DL methods are significantly small, thus confirming that these algorithms provide a good implementation strategy for embedded solutions. In fact, it can be noted that we work always with numbers represented at a single level of precision (according to IEEE754), with the memory footprints shown in Table 8.

The results are good, with only Bi-LSTM showing a higher number, while all other approaches show values that are compatible with embedded applications. The output of Bi-LSTM is due to the fact that the implementation is conceptually represented by two LSTM blocks; as a consequence, for an embedded system application, this must be carefully considered. In this specific case, the accuracy and, more generally, the performance parameters do not show a sufficient improvement to justify its adoption in a constrained environment. In contrast, approaches such as LSTM and the GRU allow for adequately precise detection capabilities with reduced consumption.

Similar considerations arise concerning an additional parameter used to evaluate the computational complexity, namely the total number of FLOPs required by each network, using the formula presented in [46] (as shown in Table 9).

Table 10 shows very good results; in fact, it shows the accuracy measured by applying the *dlQuantizer* function of MATLAB, performing network quantization. Different bit configurations were tested and the outputs were maintained for both the 16- and 32-bit configurations. This proves that the precision of the network is maintained; in addition, less memory is occupied. This is due to the fact that 16 bits occupy only half the memory of each memory cell; moreover, all configurations use integer arithmetic, with a consequent reduction in computational complexity, since, in this case, OPs are used and not FLOPs. It is worth noticing that the 8-bit configuration features a negligible accuracy drop with respect to the original network. Thus, this configuration is the most suitable for an embedded system, since it scales down the memory footprint by a factor of eight. Moreover, the computational complexity is reduced, allowing us to implement the network on very simple devices that are not equipped with a floating point unit.

The previous tables confirm LSTM as an appropriate solution for the presented work and also for similar conditions, since it combines good classification performance with a limited computational load.

An important consideration should be noted regarding falling postures, since they are the most challenging ones to detect and correctly evaluate in terms of risk: the proposed solution of radar technology combined with the LSTM or GRU allows the best identification, thus paving the way for further evaluations on larger datasets comprising a greater number of subjects who perform a broader range of movements.

The aim here was to find the most appropriate methodology; thus, in this phase, the goal was not to evaluate the performance of a specific hardware device but only to analyze the accuracy of the interface technology—in this case, MATLAB. Indeed, the characterization of the computational complexity is independent of the device that will host the code. Even so, the FLOPs represent a quantitative indicator that could be used to estimate the processing time according to the hardware characteristics.

In fact, one of the primary advantages of GRUs over LSTM and Bi-LSTM in embedded systems is their reduced computational complexity. GRUs have a simpler architecture compared to LSTM, involving fewer gates and, therefore, fewer matrix operations. This translates directly into fewer operations per time step, which is particularly beneficial for embedded systems with limited processing power. In such systems, every additional operation can significantly impact the performance, making the more efficient GRU a better choice. The reduced number of operations in a GRU allows it to be executed faster, which is critical for real-time applications where latency is a concern.

Memory is often a scarce resource on embedded systems, and minimizing the memory footprint of machine learning models is crucial. GRUs, with their simpler architecture, generally require less memory than LSTM and Bi-LSTM. This is because GRUs have fewer parameters due to the reduced number of gates and the absence of a separate cell state (which is present in LSTM).

In embedded systems, where the SRAM and DRAM capacities are limited, the lower memory requirements of GRUs allow for the more efficient use of the available resources. This not only enables the deployment of larger models within the same memory constraints but also leaves room for other essential system operations to run concurrently without exhausting the memory resources. By using GRUs, it is possible to maintain a balance between the computational performance and power efficiency, extending the operational lifespan of the device.

The inference time is the time that it takes for a model to process the input data and produce an output. In many embedded system applications, fast inference is crucial for timely decision-making. GRUs, with fewer parameters and simpler operations, generally offer faster inference times compared to LSTM and Bi-LSTM.

Embedded systems often have unique constraints and requirements that can complicate the implementation of complex neural networks. GRUs, due to their simpler structure, are easier to implement and optimize in these systems compared to LSTM and Bi-LSTM. The reduced number of parameters and operations not only simplifies the design and deployment process but also makes it easier to optimize the GRU for specific hardware architectures, leading to more efficient implementations.

Despite their simpler architecture, GRUs have been shown to offer comparable performance to LSTM in this application. In some cases, GRUs even outperform LSTM, particularly when the dataset is not too complex or when the task does not require the long-term memory capabilities that LSTM offers. This makes GRUs a more attractive option for embedded systems, where the balance between performance and resource consumption is key.

When comparing GRUs to Bi-LSTM, the performance difference can be more pronounced, especially in tasks that benefit from bidirectional processing, such as certain natural language processing applications. However, Bi-LSTM requires the processing of the data in both the forward and backward directions, effectively doubling the computational and memory requirements. For many embedded applications, the slight performance gain of Bi-LSTM does not justify the significantly higher resource consumption, making GRUs the more practical choice.

## 5. Conclusions

This paper presents an innovative solution composed of a single MIMO FMCW radar device to detect different postures and falls, whose output has been processed and classified using AI approaches in the context of ambient assisted living. The system is able to correctly detect a person in a room by removing artifacts related to the body reconstruction. The AI methodologies allow us to discriminate among positions with very good accuracy, especially the GRU and LSTM, which represent the most suitable methods for an embedded system implementation, where the resource usage could represent a potential constraint. This indicates that the proposed solution can be recommended as an approach for the evaluation of posture and falls among people at risk.

Future developments of this work will involve a larger number of healthy subjects, in order to create a larger and more representative dataset of postures, acquired with this innovative technological solution. At the same time, the AI methodologies evaluated will be fine-tuned to this context.

## Figures and Tables

**Table 1:** Mean accuracy for the three classification algorithms.

Indicator	KNN	Random Forest	SVM
Accuracy	0.958	0.941	0.931

**Table 2:** Accuracy for all AI approaches, for all training approaches.

AI Methodology	Accuracy (%)					
	50-50 Ratio	60-40 Ratio	70-30 Ratio	80-20 Ratio	90-10 Ratio	Leave-One-Out
LSTM	94.16	96.84	95.48	95.63	95.78	95.02
Bi-LSTM	96.20	96.31	96.18	96.99	95.18	96.15
Projected LSTM	96.14	96.46	96.68	96.54	97.59	96.15
GRU	95.12	95.78	95.58	95.08	97.59	95.02

**Table 3:** Precision for all AI approaches, for all training approaches.

AI Methodology	Precision (%)					
	50-50 Ratio	60-40 Ratio	70-30 Ratio	80-20 Ratio	90-10 Ratio	Leave-One-Out
LSTM	93.88	95.18	95.16	94.62	95.78	94.85
Bi-LSTM	95.39	95.61	94.85	96.52	96.22	95.05
Projected LSTM	96.40	95.20	96.30	93.98	98.30	96.88
GRU	95.01	94.20	93.93	96.77	97.79	92.59

**Table 4:** Recall for all AI approaches, for all training approaches.

AI Methodology	Recall (%)					
	50-50 Ratio	60-40 Ratio	70-30 Ratio	80-20 Ratio	90-10 Ratio	Leave-One-Out
LSTM	90.03	95.97	93.66	94.62	95.18	92.49
Bi-LSTM	96.08	95.44	92.71	96.77	94.66	94.39
Projected LSTM	95.04	94.62	95.50	96.18	95.23	95.84
GRU	89.36	88.37	90.25	92.19	91.59	92.27

**Table 5:** F1 scores for all AI approaches, for all training approaches.

AI Methodology	F1 Score (%)					
	50-50 Ratio	60-40 Ratio	70-30 Ratio	80-20 Ratio	90-10 Ratio	Leave-One-Out
LSTM	91.82	95.56	94.39	94.62	97.59	93.60
Bi-LSTM	95.72	95.52	93.75	96.64	95.38	94.71
Projected LSTM	95.70	94.91	95.90	95.05	96.70	96.35
GRU	91.84	90.52	91.98	94.35	94.26	92.43

**Table 6:** Area under the curve for all AI approaches, for all training approaches.

AI Methodology	Area under the Curve (AUC—%)					
	50-50 Ratio	60-40 Ratio	70-30 Ratio	80-20 Ratio	90-10 Ratio	Leave-One-Out
LSTM	98.89	99.43	99.52	99.47	99.25	98.21
Bi-LSTM	99.26	99.35	99.10	99.61	99.51	98.70
Projected LSTM	99.44	99.53	99.68	99.58	99.67	99.54
GRU	99.18	99.34	98.95	99.38	99.81	98.44

**Table 7:** Iterations per epoch to reach maximum accuracy.

AI Methodology	Iterations per Epoch					
	50-50 Ratio	60-40 Ratio	70-30 Ratio	80-20 Ratio	90-10 Ratio	Leave-One-Out
	51	62	72	83	93	90

**Table 8:** Memory footprints for all AI approaches.

AI Methodology	Memory Footprint (Bytes)
LSTM	172,400
Bi-LSTM	344,800
Projected LSTM	192,000
GRU	129,600

**Table 9:** Total FLOPs for all AI approaches.

AI Methodology	FLOPs
LSTM	13,477,828
Bi-LSTM	52,235,628
Projected LSTM	13,477,828
GRU	7,947,400

**Table 10:** Accuracy of the network with the 80-20 ratio with different bit configurations.

AI Methodology	Accuracy (%)			
	8 (4.4) Bits	16 (8.8) Bits	32 (16.16) Bits	Floating Point
LSTM	95.18	95.63	95.63	95.63
Bi-LSTM	96.99	96.99	96.99	96.99
Projected LSTM	97.59	96.54	96.54	96.54
GRU	95.93	96.08	96.08	96.08

## Data Availability

The raw data supporting the conclusions of this article will be made available by the authors on request.
